# Concrete Structure Inspired *3D‐Printed* Framework Mechanically Reinforced Zwitterionic Hydrogel for Efficient Postoperative Abdominal Adhesion Prevention

**DOI:** 10.1002/advs.202508954

**Published:** 2025-09-16

**Authors:** Jianguo Song, Xin Cao, Ruirui Zhang, Shujie Cheng, Yi Wang

**Affiliations:** ^1^ College of Mechanical & Energy Engineering Beijing University of Technology Beijing 100124 China; ^2^ Basic Research Key Laboratory of General Surgery for Digital Medicine Affiliated Hospital of Hebei University Baoding 071000 China

**Keywords:** 3D printing, abdominal adhesion prevention, concrete‐inspired, framework‐reinforced structure, lubricating hydrogel

## Abstract

Zwitterionic hydrogels, which have been demonstrated to hold great potential in preventing abdominal adhesion, are hindered in clinical translation by their inherent mechanical fragility and structural instability. Inspired by the framework‐reinforced composite structure of concrete in building construction, herein, a 3D‐printed polymeric framework reinforced zwitterionic lubricating hydrogel (abbreviated as 3DF‐LH) is developed, which features high tensile strength internally and optimal lubrication externally. Just like the establishment process of concrete, 3DF‐LH is fabricated by infiltrating hydrogel precursor solution, that consisted of GelMA and zwitterionic sulfobetaine methacrylate (SBMA), into the pores of a 3D‐printed PCL framework (3DF), followed by UV curing. In vitro studies demonstrated that the 3DF‐LH composite exhibited superior structural stability, mechanical robustness, and biocompatibility, along with significantly suppressing fibroblast adhesion. In an SD rat cecal‐abdominal wall postoperative adhesion model, 3DF‐LH attenuated the inflammatory microenvironment, which involved the suppression of the TGF‐β/Smad signaling pathway, thereby effectively inhibiting abdominal adhesion formation. These findings demonstrate that developed 3DF‐LH effectively addresses the mechanical limitations of zwitterionic hydrogels and thus holds great potential for future clinical translation.

## Graphical abstract

Inspired by the establishment process and structure of concrete, a 3D‐printed PCL framework‐reinforced composite hydrogel is developed to overcome the mechanical limits of the original zwitterionic hydrogel toward clinic application of postoperative adhesion prevention.

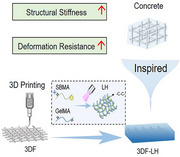

## 1 Introduction

Abdominal adhesions represent the most prevalent postoperative complication following abdominal procedures, predisposing patients to lifelong clinical sequelae, including chronic pelvic pain, female infertility, intestinal obstruction, and mortality risks.^[^ ^1^, ^2^, ^3^, ^4^ ^]^ Conventional injectable hydrogels face a fundamental trade‐off where enhancing mechanical properties often compromises biocompatibility or interfacial lubrication. For instance, Wang et al.’s hydrogen‐bond‐interlocked polyurethane hydrogel achieved high strength but raised concerns over catalyst residues;^[^ ^5^ ^]^ Ling et al.’s mechanically trained hydrogel exhibited degradation heterogeneity due to mechanical anisotropy, limiting uniform barrier function;^[^ ^6^ ^]^ and Wu et al.’s sprayable HADP hydrogel enabled “internal adhesion‐external lubrication” yet required invasive photoactivation.^[^ ^7^ ^]^ While these innovations address specific challenges, their limitations in achieving simultaneous mechanical robustness, biocompatibility, and surgical practicality hinder injectable systems from supplanting solid barriers in clinical practice.^[^ ^8^ ^]^ Among solid alternatives, hyaluronic acid (HA) membranes prevent adhesions via interfacial lubrication but suffer from intrinsic mechanical fragility. This vulnerability is exemplified by pure HA gels displacing under physiological stress in rat models, which necessitates reinforcement strategies such as keratinocyte growth factor (KGF) nanoparticles for functional stability.^[^ ^9^ ^]^

Notably, the interfacial lubrication that underlies the anti‐adhesive function of materials like HA membranes aligns with a more fundamental biological mechanism: hydration lubrication. This process relies on zwitterionic polymers forming bound hydration shells through ion‐dipole interactions to deliver essential anti‐adhesive effects.^[^ ^10^ ^]^ However, this mechanism critically depends on interfacial hydration dynamics,^[^ ^11^, ^12^ ^]^ rendering lubricious hydrogels inherently susceptible to structural failure under physiological stress. Our prior work confirmed such vulnerability: hydrated lubricant surfaces reduced adhesion in vivo but suffered mechanical compromise under dynamic abdominal forces.^[^ ^13^ ^]^

**Figure 1:**
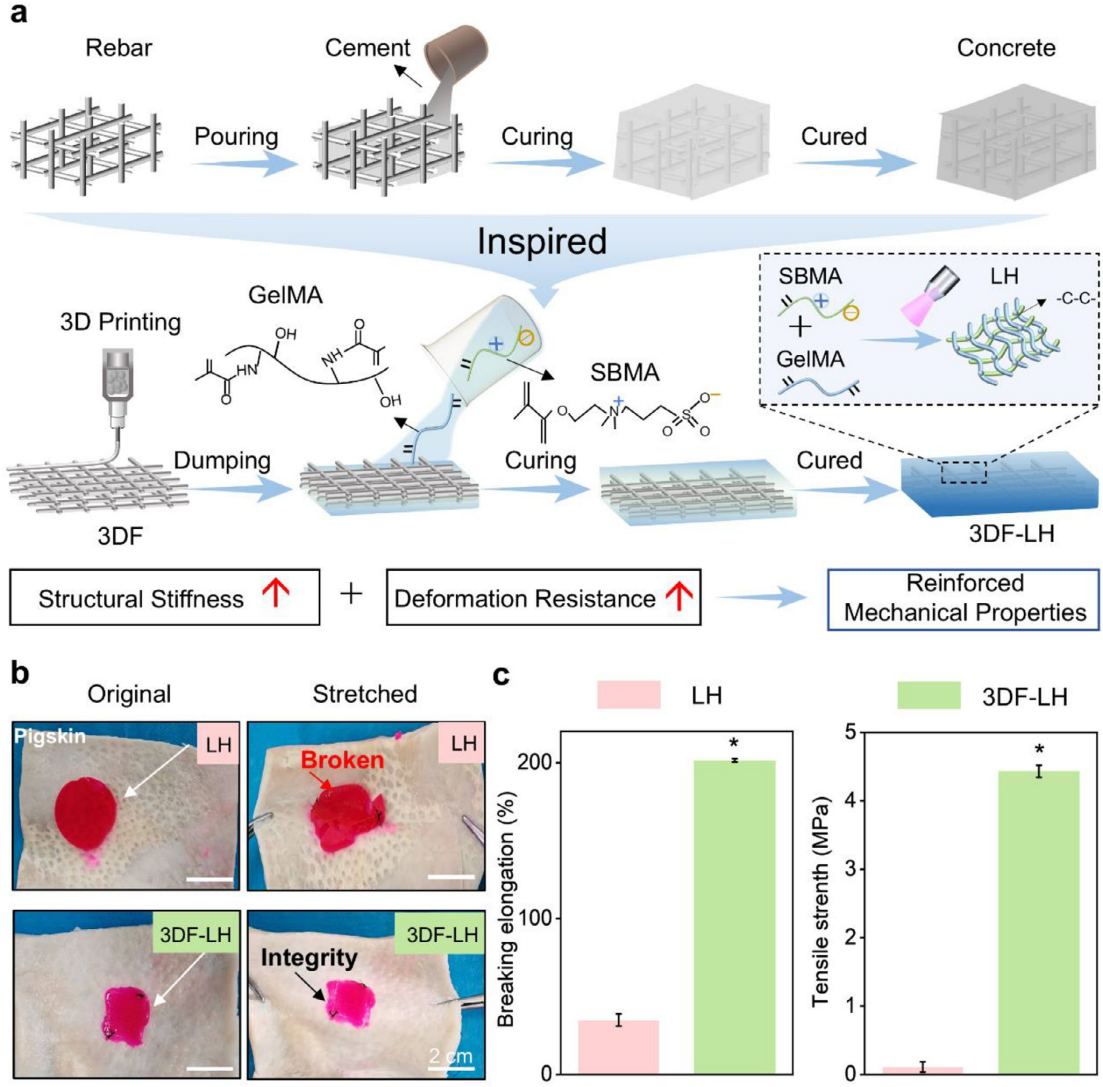
Our developed concrete‐inspired 3DF‐LH effectively addresses the mechanical problem of zwitterionic hydrogels in clinical applications. a) Schematic diagram of the 3DF‐LH preparation process. b) Comparison of mechanical properties between LH and 3DF‐LH in an in situ pigskin stretching experiment. c) Tensile strength and breaking elongation of LH/3DF‐LH. Data are presented as mean ± SDs, ^*^ *p* < 0.05 compared to the 3DF‐LH group.

Inspired by concrete's structural reinforcement (**Figure** **1**a), we engineered a “concrete‐mimetic” composite hydrogel to overcome the inherent mechanical limitations of zwitterionic hydrogels. This system integrates a 3D‐printed PCL framework (structural rebar analogue) with a lubricating matrix (concrete analogue). The 3D PCL porous framework enabled simultaneous mechanical reinforcement and controlled hydrogel permeation. This architectural integration conferred a load‐bearing core while maintaining a lubricious boundary layer, creating a unique duality: structural rigidity prevented suture pull‐through during fascial closure, while surface hydration lubrication facilitated organ gliding during peritoneal movement. Physiomimetic testing under cyclic abdominal wall loading revealed that the framework‐reinforced lubricating hydrogel (3DF‐LH) exhibited significantly enhanced mechanical properties compared to the unreinforced lubricating hydrogel (LH), achieving a tensile strength of 4.43 ± 0.14 MPa and breaking elongation of 205.14 ± 3.23% (Figure 1b). The framework‐hydrogel interface effectively prevented stress concentration‐induced matrix failure during dynamic deformation. Crucially, the integrated design preserved structural integrity beyond clinically relevant loading cycles while maintaining adequate suture retention forces for in vivo fixation requirements.

3DF‐LH was constructed through the photopolymerization of GelMA and SBMA within the microarchitecture of a 3D‐printed PCL framework. To dissect the role of individual components, two control groups were established: 3D‐printed PCL framework (3DF) and 3D framework‐reinforced hydrogel (3DF‐H). Subsequently, in vitro tensile properties, compressive properties, swelling properties, antibacterial adhesion properties, hemostatic characteristics, cellular activity, hemocompatibility, and degradation properties of 3DF, 3DF‐H, and 3DF‐LH were investigated. In addition, an SD rat cecum‐abdominal wall‐adhesion model was established to evaluate 3DF‐LH's anti‐adhesion and anti‐inflammatory effects and to explore the corresponding mechanisms. We hypothesize that 3DF‐LH overcomes postoperative abdominal adhesions via dual synergistic mechanisms: mechanical reinforcement and interfacial lubrication, integrating structural stability with anti‐adhesive functionality.

## 2 Results and Discussion

### 2.1 Fabrication and Characterization of 3DF‐LH

**Figure 2:**
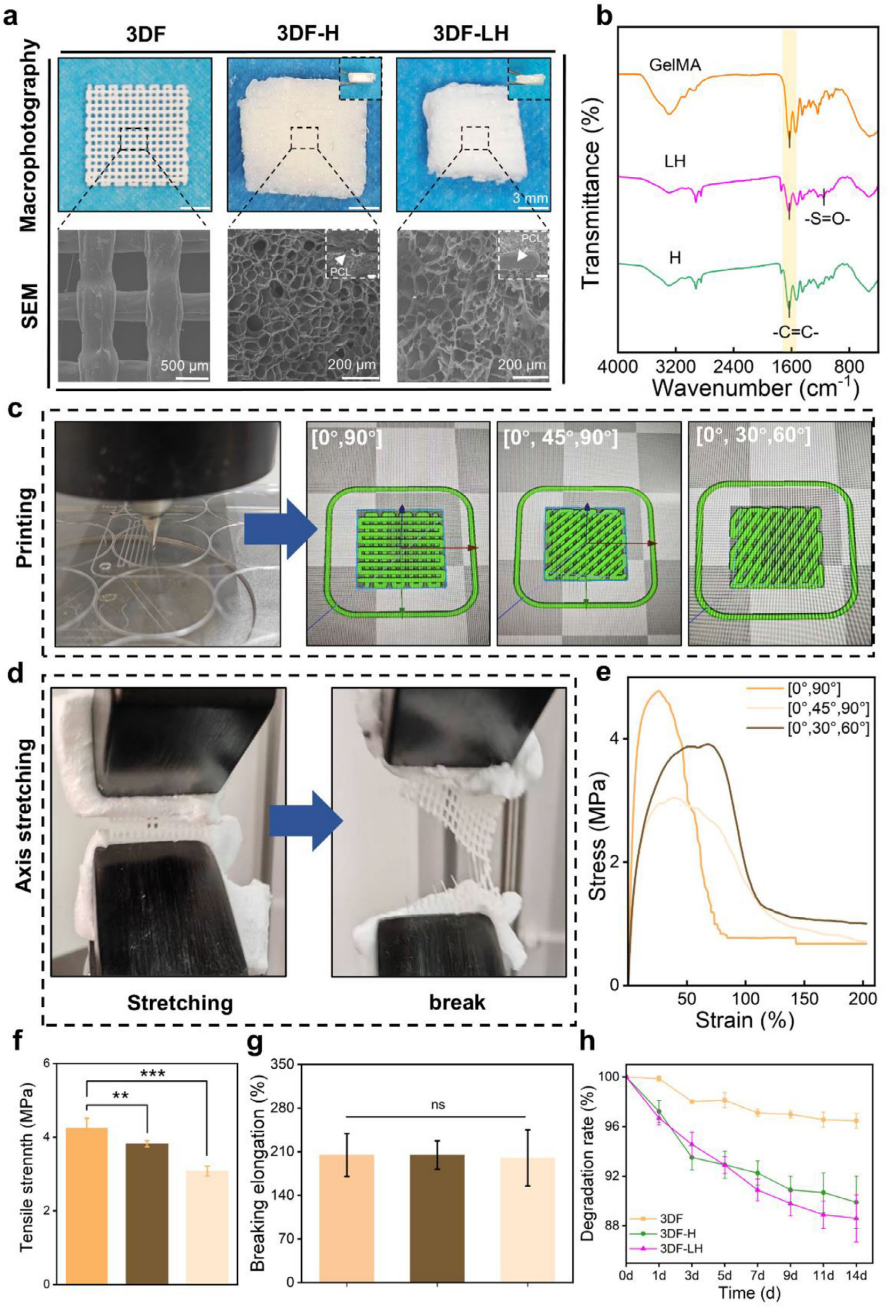
Material characterizations. a) Macroscopic photos and microscopic SEM images of the 3DF, 3DF‐H, and 3DF‐LH groups. b) FTIR transmittance spectra of the GelMA, LH, and H groups. c) Photos of the printed frameworks with different designed patterns. d) Demonstration of framework stretching from intact to fractured states. e) Stress‐strain curves of frameworks in different designed patterns. f) Tensile strength and g) Elongation at break of frameworks in different designed patterns. h) Degradation rate curves of 3DF, 3DF‐H, and 3DF‐LH frameworks in PBS solution supplemented with collagenase II. Data are presented as mean ± SDs, ^**^ *p* < 0.01, ^***^ *p*< 0.001, ns: no significance.

The 3DF‐LH was fabricated by encapsulating 3D‐printed PCL frameworks with photopolymerized GelMA and SBMA hydrogels, while the 3DF‐H was fabricated by encapsulating 3D‐printed PCL frameworks with GelMA hydrogel. Macroscopic evaluation revealed complete encapsulation of the framework by the lyophilized hydrogel matrix. SEM images of lyophilized 3DF, 3DF‐H, and 3DF‐LH (**Figure** **2**a) demonstrated distinct microstructural architectures: Both the 3DF‐LH and 3DF‐H groups exhibited uniformly porous hydrogel network structures. Further evaluation is warranted to determine whether 3DF‐LH can effectively maintain a moist wound environment while enabling oxygen permeability—properties critical for clinical translation.^[^ ^14^, ^15^ ^]^ To evaluate this key functionality, functional characterization of 3DF‐LH demonstrated that its Water Vapor Transmission Rate (WVTR) and Oxygen Permeability Coefficient (OPC) significantly surpass those of the commercial product Tegaderm, confirming its superior clinical applicability (Figure S1, Supporting Information). These enhanced WVTR and OPC properties facilitate nutrient/metabolite exchange while promoting the recruitment of inflammatory cells (e.g., macrophages), thereby establishing optimal conditions for tissue regeneration.^[^ ^16^, ^17^ ^]^

Fourier‐transform infrared spectroscopy results confirmed successful chemical integration of SBMA into the GelMA network (Figure 2b). Post‐photopolymerization spectra showed significant attenuation of the C═C bond stretching peak at 1630–1640 cm^−1^,^[^ ^18^ ^]^ accompanied by the emergence of a sulfonic group vibration peak at 1151 cm^−1^,^[^ ^19^ ^]^ verifying covalent incorporation of zwitterionic SBMA moieties. Physiological stress events such as sneezing or coughing in healthy individuals can generate transient peak intra‐abdominal pressures (IAP) reaching 0.02 MPa.^[^ ^20^ ^]^ Effective peritoneal adhesion prophylaxis devices must demonstrate biomechanical resilience to withstand these maximal IAP thresholds while preserving structural integrity and retaining more than 90% of initial tensile strength post‐exposure to simulated physiological pressure. By adjusting the line design in 3D printing process, three distinct configurations, 0°‐90°, 0°‐45°‐90°, and 0°‐30°‐60°, were fabricated (Figure 2c). Tensile testing revealed significant anisotropic mechanical properties across all scaffold types (Figure 2d). All groups exhibited tensile strengths exceeding 3 MPa (Figure 2e), substantially surpassing the requirements for abdominal anti‐adhesion barriers. The 0°‐90° configuration demonstrated maximal tensile strength of 4.30 ± 0.34 MPa (Figure 2f), while maintaining comparable fracture elongation rates of ≈208.03 ± 31.11% (Figure 2g). Additionally, compression testing confirmed that the 0°‐90° configuration exhibited the highest compressive modulus, reaching 9.30 ± 0.09 MPa (Figure S2a, Supporting Information). This optimized line design achieved an ideal balance between stiffness and deformation resistance, ensuring favorable conformability to abdominal tissues while providing robust protection against potential organ‐surface exposure. Based on these findings, the 0°‐90° patterned framework was selected for subsequent experimental investigations.

### 2.2 In Vitro Degradation and Swelling Profile

Material degradability represents a fundamental characteristic of implantable bio‐scaffolds,^[^ ^21^ ^]^ as physical barriers with short residence time demonstrate insufficient efficacy in preventing postoperative adhesion.^[^ ^22^ ^]^ To evaluate degradation behavior, 3DF, 3DF‐H, and 3DF‐LH samples were subjected to gravimetric analysis in collagenase II‐supplemented PBS over 14 days. All groups retained over 88% of their initial mass at the endpoint (Figure 2h), with 3DF‐LH demonstrating superior structural stability (89.4 ± 2.1% mass retention). This sustained integrity during the critical adhesion formation window (postoperative days 3–5)^[^ ^23^ ^]^ ensures uninterrupted barrier functionality, thereby addressing the clinical limitation of premature dissolution observed in conventional physical barriers (e.g., hyaluronic acid films, which degrade within 48–72 h).

Beyond degradation resistance, suppressing swelling is also essential for effective anti‐adhesion barriers. Mechanistically, zwitterionic groups orchestrate dense hydrogen‐bonding networks with water molecules via electrostatic attraction, and these networks ensconce the gel matrix in stable hydration layers. This molecular architecture kinetically impedes free‐water diffusion into the network, thereby suppressing equilibrium swelling.^[^ ^24^ ^]^ Consistent with this mechanism, the 3DF‐LH exhibited outstanding anti‐swelling efficacy after 48 h immersion in PBS, as evidenced by its low swelling ratio (Figure S2b, Supporting Information). Despite demonstrating accelerated swelling kinetics relative to 3DF‐H (reaching equilibrium within 24 h—twice as rapidly), 3DF‐LH achieved a significantly lower equilibrium swelling ratio (8.81 ± 1.50), representing a 46% reduction compared to 3DF‐H.

### 2.3 Biocompatibility Evaluation

**Figure 3:**
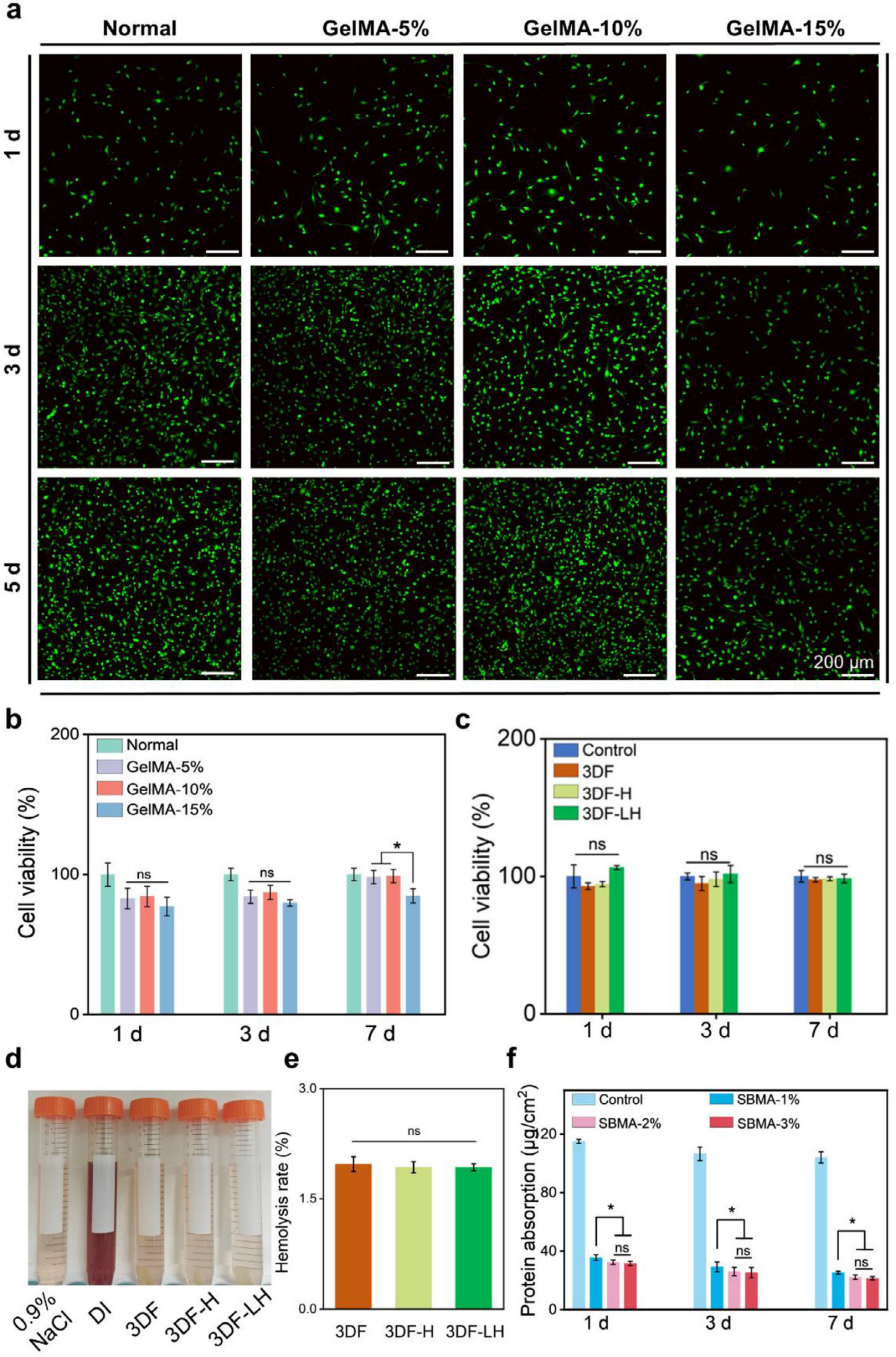
In vitro biocompatibility and anti‐protein adsorption study. a) Representative live/dead staining images of NIH/3T3 cells cultured with different GelMA hydrogels at days 1, 3 and 7. b) Quantifications of cellular viability of NIH‐3T3 cells cultured with different GelMA hydrogels at days 1, 3 and 7. c) Quantifications of cellular viability of NIH‐3T3 cells cultured with control, 3DF, 3DF‐H, and 3DF‐LH at days 1, 3 and 7. d) Photographic documentation of hemolysis rates across experimental groups. e) Quantitative analysis of hemolysis rates for 3DF, 3DF‐H, and 3DF‐LH. f) Comparison of protein adsorption resistance among different SBMA concentrations. Data are presented as mean ± SDs, ^*^ *p* < 0.05, ns: no significance.

Cytocompatibility represents the paramount prerequisite for hydrogel‐based anti‐adhesion barriers in clinical applications.^[^ ^25^ ^]^ PCL, GelMA, and SBMA were selected based on their reported biocompatibility profiles.^[^ ^26^ ^]^ NIH/3T3 fibroblast viability was evaluated using live/dead staining and CCK‐8 assays. Live/dead imaging (**Figure** **3**a) demonstrated significantly higher viable cell densities in the 10% GelMA‐3DF‐LH group compared to 5% and 15% GelMA formulations on days 3 and 7, with minimal dead cells observed across all groups. CCK‐8 quantification (Figure 3b) corroborated these findings, showing superior cell viability in the 10% GelMA group at day 7 compared to 5% and 15% groups (p < 0.05). This superiority of the 10% GelMA formulation stems from its balanced porous structure and mechanical properties: unlike the 15% GelMA, which exhibits excessive crosslinking (Figure S3a, Supporting Information), reduced porosity (Figure S3b, Supporting Information), and overly high compressive modulus (impairing cell viability), and contrasting with the 5% GelMA that shows suboptimal mechanical strength (Figure S3c, Supporting Information), elevated residual cytotoxic monomers from incomplete polymerization,^[^ ^27^ ^]^ and accelerated degradation overwhelming cellular clearance to cause microenvironmental imbalance, the 10% GelMA provides favorable conditions for cell viability. Consequently, the 10% GelMA concentration was selected as the optimal formulation for subsequent experiments. To further validate biocompatibility, CCK‐8 assays were performed to assess cell viability across control, 3DF, 3DF‐H, and 3DF‐LH groups. All the samples revealed no significant differences in cell viability from day 1 to day 7, which further indicated that the 3DF‐LH was not cytotoxic to NIH/3T3 cells in vitro (Figure 3c). Hemocompatibility, a critical determinant of biomaterial safety,^[^ ^28^ ^]^ was assessed via standardized hemolysis assays. As Figure 3d shows, severe hemolysis was observed in the Triton‐X‐100 positive control group, characterized by a marked color transition from colorless to bright red in the supernatant. Both negative controls (PBS) and experimental groups (3DF, 3DF‐H, 3DF‐LH) exhibited negligible hemolytic activity, as evidenced by the absence of pronounced colorimetric changes (pale yellow supernatant). Hemolysis quantification (Figure 3e) confirmed compliance with international biomedical material standards, with all test groups maintaining hemolysis rates below the 5% critical threshold.^[^ ^29^ ^]^ This dual validation through visual observation and quantitative metrics substantiates their clinical applicability in blood‐contacting environments. These results collectively demonstrate the absence of cytotoxic effects on cellular proliferation and confirm favorable in vitro biocompatibility across all groups.

### 2.4 In Vitro Anti‐Adhesion Efficacy Evaluation

**Figure 4:**
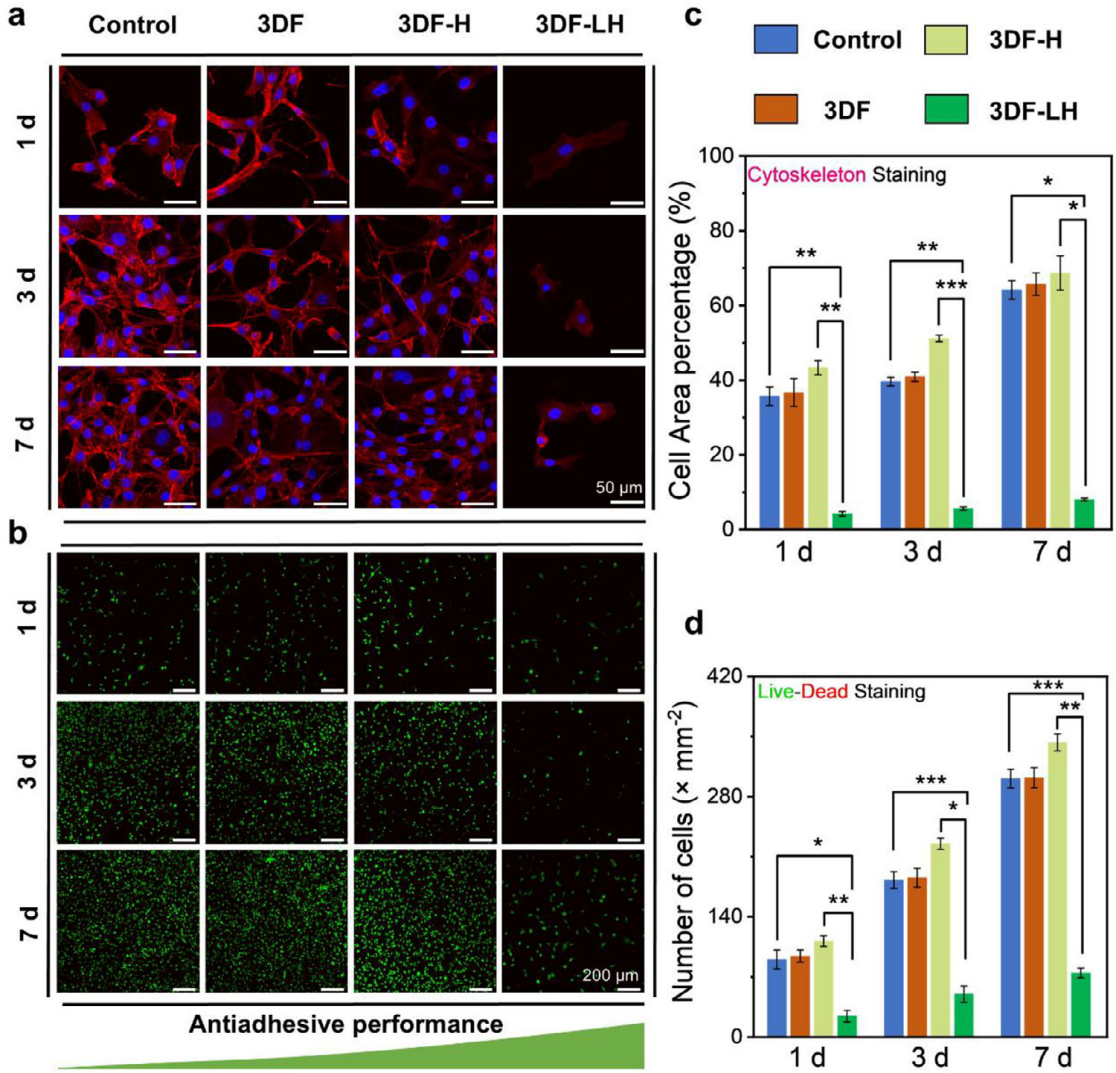
In vitro anti‐adhesion efficacy evaluation. a) Phalloidin staining of the cytoskeleton showing cell adhesion on Control, 3DF, 3DF‐H, and 3DF‐LH at days 1, 3, and 7. b) Live/dead staining images on Control, 3DF, 3DF‐H, and 3DF‐LH at days 1, 3, and 7. Quantitative analysis of c) cell adhesion area percentage and d) adherent cell counts for Control, 3DF, 3DF‐H, and 3DF‐LH at days 1, 3, and 7. Data are presented as mean ± SDs, ^*^ *p* < 0.05, ^**^ *p* < 0.01, ^***^ *p* < 0.001.

Quantitative protein adsorption analysis revealed concentration‐dependent anti‐fouling efficacy of zwitterionic SBMA modification. Both 2% and 3% SBMA formulations demonstrated no statistically significant difference in anti‐fouling performance (2%: 36.14 ± 2.80 µg cm^−2^ vs 3%: 34.92 ± 3.11 µg cm^−2^; p = 0.32), both of which significantly outperformed the 1% SBMA group (38.45 ± 5.61 µg cm^−2^; p < 0.001 compared to the 1% group) at day 7 (Figure 3f). Despite the comparable protein resistance achieved with 2% and 3% SBMA concentrations, the 2% formulation was ultimately selected for 3DF‐LH synthesis based on its optimal balance of anti‐fouling and mechanical properties. This strategic optimization balances interfacial anti‐adhesion capability with structural integrity requirements for surgical handling. Given that postoperative adhesion represents a potential pathological outcome of wound healing,^[^ ^30^ ^]^ characterized by impaired fibrin clearance at surgical sites leading to persistent fibrin matrices that facilitate excessive fibroblast proliferation and collagen deposition, ultimately progressing to fibrotic adhesions,^[^ ^31^ ^]^ we evaluated fibroblast adhesion. Cytoskeletal analysis revealed significantly reduced NIH/3T3 adhesion onto 3DF‐LH, as evidenced by adherent cell areas that were 33.41% (day 1), 35.14% (day 3), and 61.33% (day 7) smaller than those on other groups (**Figure** **4**a,c). Live/dead assays corroborated these findings, revealing a significantly lower cell density on 3DF‐LH (67.90 ± 7.21 cells mm^−^^2^) compared to the control group (313.15 ± 21.31 cells mm^−^^2^) at day 7 (p < 0.05; Figure 4b,d). Our in vitro investigations demonstrated that 3DF‐LH enhanced anti‐adhesion efficacy by stabilizing zwitterionic hydration lubrication mechanisms, leading to significant reductions in fibroblast adhesion and proliferation.

### 2.5 In Vivo Anti‐Bacterial Adhesion Efficacy

During abdominal surgery, bacterial adherence to the surgical wound surface can lead to biofilm formation, triggering local infection.^[^ ^32^ ^]^ Simultaneously, bacteria can secrete an extracellular polymeric substance (EPS) matrix, promoting fibrin deposition and fibroblast proliferation, which directly exacerbates tissue adhesion.^[^ ^33^ ^]^ Consequently, we also evaluated the anti‐bacterial adhesion and bactericidal properties of 3DF‐LH. Bacterial adhesion to the hydrogels to Gram‐positive and Gram‐negative bacteria was, respectively, assessed by incubating hydrogels with *S*taphylococcus aureus *(S. aureus)* and Escherichia coli *(E. coli)* for 1 h. The excellent antibacterial effect of 3DF‐LH was validated by plate counting (Figure S4a, Supporting Information). The quantitative Colony‐Forming Unit (CFU) counts were as follows: for *S. aureus*, 3554.31 ± 52.00 (Blank), 3364.31 ± 149.35 (3DF), 3398.01 ± 190.59 (3DF‐H), and 17.31 ± 5.44 (3DF‐LH); for *E. coli*, 3242.00 ± 91.41 (Blank), 3179.36 ± 154.05 (3DF), 3015.07 ± 83.67 (3DF‐H), and 33.00 ± 4.00 (3DF‐LH) (Figure S4b, Supporting Information). These results suggest that 3DF‐LH likely prevents the initial bacterial adhesion via a surface hydration layer.

The antibacterial activity was assessed using live/dead staining, where live bacteria exhibited green fluorescence and dead bacteria exhibited red fluorescence. As shown in Figure S4c (Supporting Information), 3DF‐LH showed almost entirely red fluorescence caused by the antibacterial action of zwitterionic groups. Microscopic morphology of bacteria after different treatments was observed using SEM (Figure S4d, Supporting Information). After 3DF and 3DF‐H treatment, both *S. aureus* and *E. coli* preserved an intact cell membrane with a smooth surface. Conversely, the 3DF‐LH group exhibited the most significant lesions and atrophy, and the dead bacteria would drop off from the surface. Hence, 3DF‐LH exhibited excellent antibacterial activity with high efficiency for application in the treatment of abdominal cavity adhesions.

### 2.6 In Vitro and Vivo Hemostatic Properties

Excellent hemostatic performance can significantly enhance the prevention effect of anti‐adhesion materials on postoperative adhesions.^[^ ^34^ ^]^ First, as shown in Figure S5a (Supporting Information), the hemostatic capacity of 3DF‐LH was evaluated in the liver model, which contains the most abundant blood vessels among visceral organs. Blood gushed out once the wound was created, and impregnated the entire gauze in the control and 3DF groups. In contrast, the areas of blood staining were smaller in the 3DF‐H and 3DF‐LH groups, with 3DF‐LH exhibiting minimal staining among all groups. In addition, the blood loss was in the following order (Figure S5b, Supporting Information): control group (4.05 ± 0.23 g) > 3DF group (4.04 ± 0.15 g) > 3DF‐H group (3.67 ± 0.61 g) > 3DF‐LH group (1.48 ± 0.60 g).

The blood clotting index (BCI) was also used for a preliminary assessment of the hemostatic capacity of 3DF‐LH. As shown in Figure S5b (Supporting Information), the quantitative values in the control, 3DF, 3DF‐H, and 3DF‐LH groups reached 108.51 ± 1.43%, 98.51 ± 2.90%, 33.86 ± 7.4% and 11.99 ± 3.18%, respectively, suggesting the excellent blood clotting capacity of 3DF‐LH.

### 2.7 In Vivo Anti‐Adhesion Efficacy and Mechanistic Exploration

**Figure 5:**
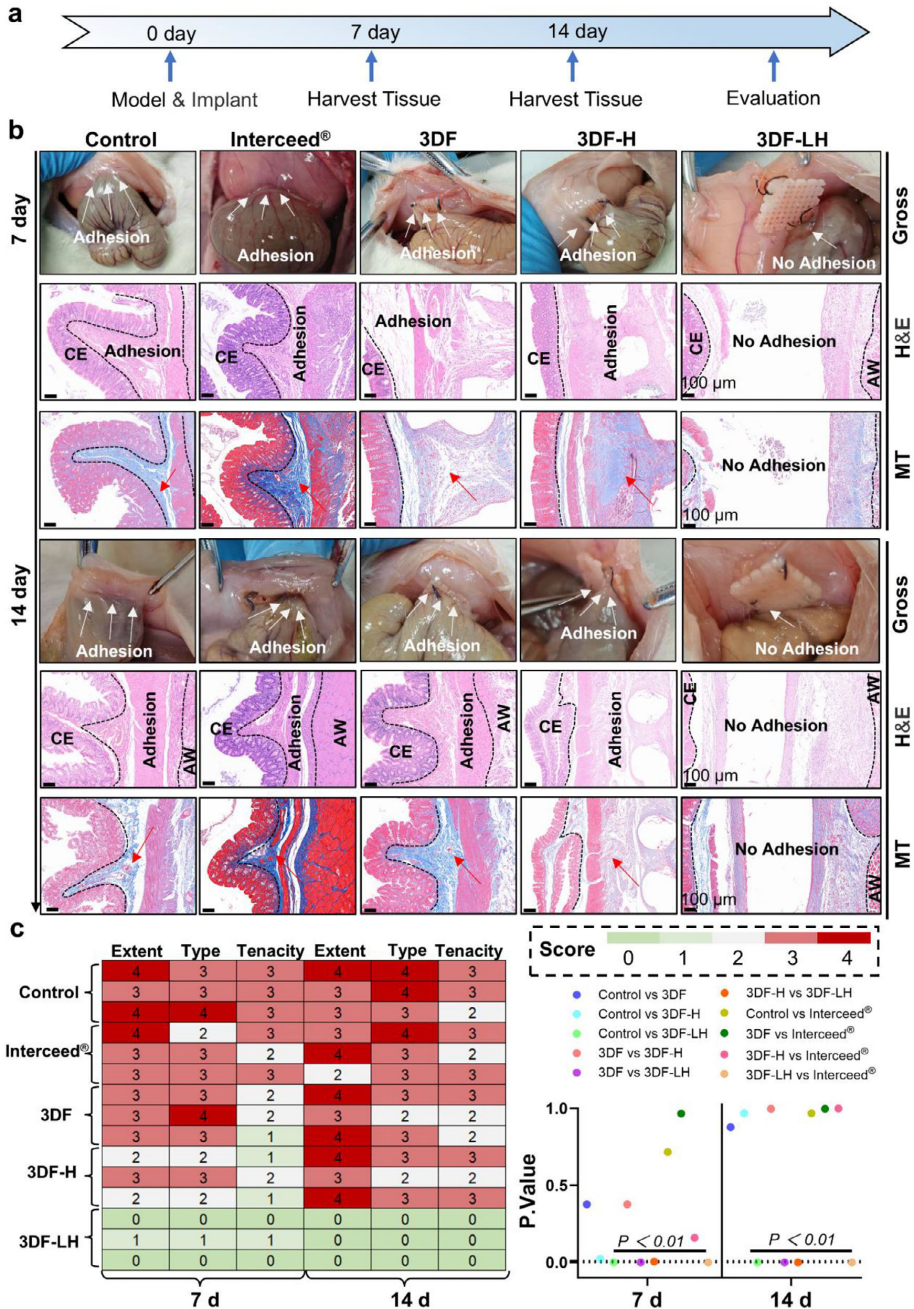
In vivo anti‐adhesion evaluation based on a Sprague‐Dawley (SD) rat abdominal model. a) Experimental schedule of in vivo anti‐adhesion assessment. b) Representative photographs of abdominal adhesions in rats after different treatments, and H&E and Masson staining results of adhesion tissues collected on postoperative days 7 and 14. CE: cecum, AW: abdominal wall. c) Quantification of adhesion scores in rats across treatment groups on postoperative days 7 and 14, with pairwise comparison of p‐values among the Control, Interceed^®^, 3DF, 3DF‐H, and 3DF‐LH groups.

**Figure 6:**
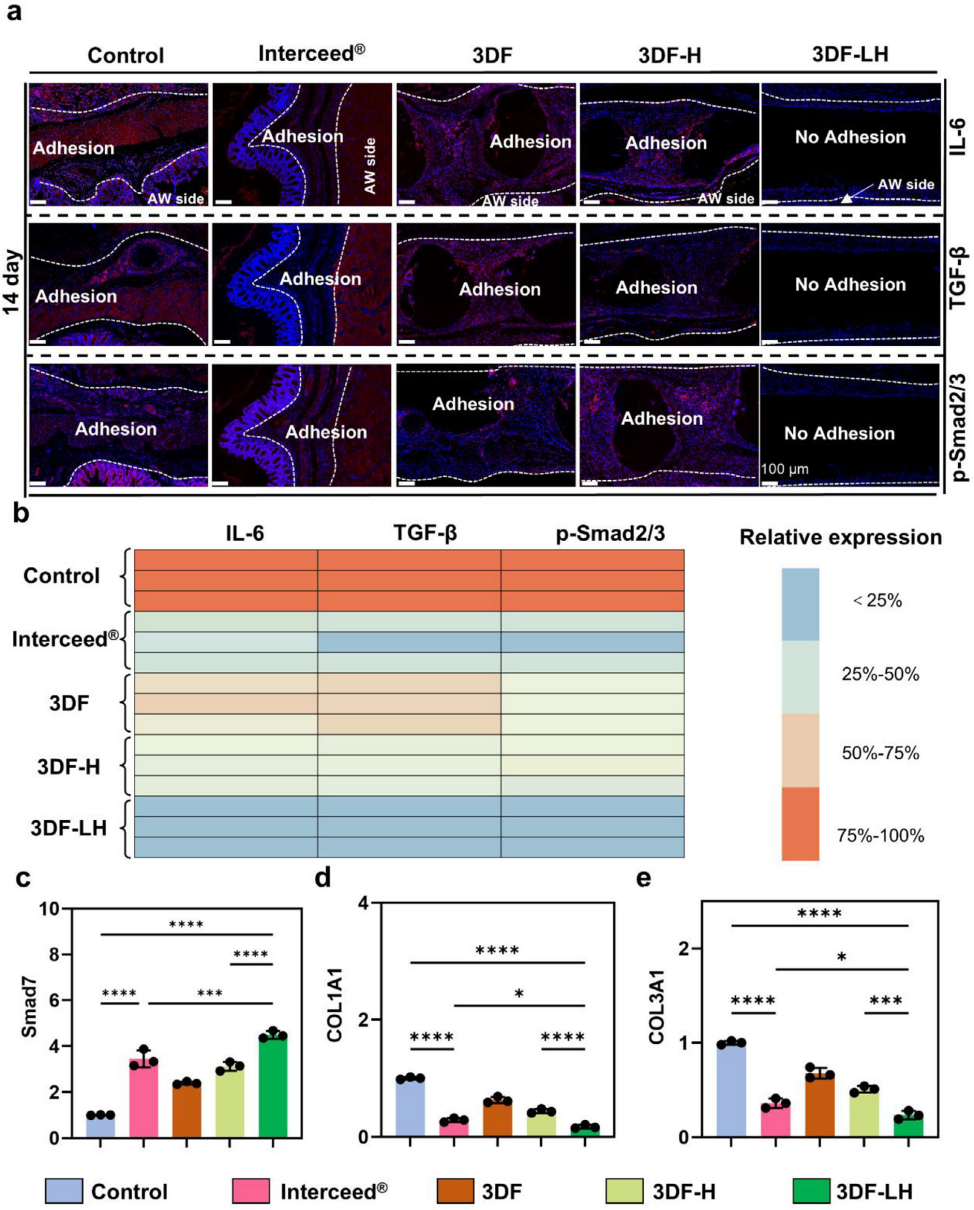
Mechanistic investigation of 3DF‐LH on anti‐adhesion performance. a) Representative images of immunofluorescence staining for IL‐6, TGF‐β, and P‐Smad2/3 on day 14. b) Mean fluorescence intensity of IL‐6, TGF‐β, and P‐Smad2/3 on day 14, respectively. mRNA levels of Smad7 c), COL1A1 d), and COL3A1 e) in tissues after different treatments on day 14. Data are presented as mean ± SDs, ^*^ *p* < 0.05, ^***^ *p* < 0.001, ^****^ *p* < 0.0001.

To validate the anti‐adhesion efficacy of 3DF‐LH, we established a rat cecal‐abdominal wall abrasion model (**Figure** **5**a), which is a well‐established preclinical system for evaluating adhesion barriers. Untreated rats served as negative controls, and postoperative adhesion severity across all groups was systematically evaluated at Day 7 and 14 using a standardized adhesion scoring system. This system comprehensively assessed three critical parameters: the extent of the involved anatomical area, the morphological type of adhesion, and the mechanical tenacity of adhesions. As shown in Figure 5b,c, all untreated controls developed severe adhesions (mean score: 3.44 ± 0.38) by Day 7, confirming successful model induction. While Interceed, 3DF, and 3DF‐H groups showed limited efficacy (no scores < 2), 3DF‐LH significantly reduced adhesion formation (mean score: 0.47 ± 0.20 vs controls, p < 0.001), with complete prevention observed in 67% of cases. At 14 days post‐implantation, rats in the control, 3DF, and 3DF‐H groups exhibited exacerbated adhesion formation, evidenced by elevated mean adhesion scores. Concurrently, Interceed demonstrated efficacy, with the adhesion score reduced to 1.41 ± 0.20. In stark contrast, three rats in the 3DF‐LH cohort displayed no macroscopic adhesions (score: 0). As shown in **Figure** **6**C, pairwise comparisons among Control, Interceed, and 3DF‐LH groups demonstrated statistically significant differences in adhesion scores (P < 0.01 for all pairs). The experimental findings demonstrate that pure PCL frameworks inherently lack anti‐adhesive properties. In contrast, the composite scaffold system integrating GelMA and SBMA exhibits remarkable therapeutic efficacy against postoperative adhesion, surpassing even the commercial product Interceed. This bioinspired composite architecture follows a reinforcement principle analogous to steel‐concrete synergy in construction‐GelMA establishes an ECM‐mimetic bioactive interface to promote cellular interaction, while SBMA creates a zwitterionic anti‐fouling barrier through its charge‐balanced hydration layer. Their synergistic interplay significantly reduces protein adsorption and subsequent fibroblast attachment, thereby effectively mitigating tissue adhesion risks through both biomimetic integration and molecular‐level repulsive mechanisms. H&E and Masson staining images (Figure 5b) revealed distinct tissue responses. Untreated controls exhibited dense fibrotic adhesions with disorganized collagen deposition at day 7, progressing to vascularized, collagen‐rich lesions at day 14. Animals treated with the commercial barrier Interceed showed moderate improvement characterized by reduced fibrosis and partial collagen organization at both timepoints. In contrast, 3DF‐LH‐treated rats showed minimal connective tissue at day 7 and near‐complete mesothelial regeneration at day 14, indicating active tissue repair.^[^ ^35^ ^]^ Histopathological examination of representative vital organs (hearts, livers, spleens, lungs, kidneys) and surgical suture site tissues harvested at 14 days post‐implantation confirmed systemic biocompatibility, revealing intact tissue architecture with no evidence of necrosis, inflammation, or fibrosis (Figure S6, Supporting Information). These findings were corroborated by comprehensive hematological analysis (complete blood count) and serum biochemical markers (ALT, AST, BUN, Crea), all demonstrating values within normal physiological ranges, suggesting intact hepatic and renal function (Table S1, Supporting Information). Collectively, through multimodal analyses, we validated the optimal biocompatibility of 3DF‐LH.

In the early stage of abdominal wall and cecal trauma, immune cells, including macrophages, infiltrate the wound site and release inflammatory factors.^[^ ^36^ ^]^ This inflammatory microenvironment constantly disrupts the homeostasis of the fibrinolytic system,^[^ ^37^ ^]^ eventually leading to adhesion formation. Studies have shown that TGF‐β1, as one of the most important triggering factors in tissue fibrotic lesions,^[^ ^38^ ^]^ can stimulate peritoneal mesothelial cells to secrete collagen and inhibitors of matrix protein‐degrading enzymes.^[^ ^39^ ^]^ Therefore, we used immunofluorescence staining to quantitatively evaluate inflammatory cytokines in vivo, including IL‐6, TGF‐β1, and P‐Smad2/3 (Figure 6a). The quantitative analysis results (Figure 6b) showed that the control group exhibited significantly higher expression levels of IL‐6, TGF‐β1, and p‐Smad2/3 in the adhesion zone and surrounding areas. However, the 3DF, 3DF‐H, Interceed, and 3DF‐LH groups all showed relatively low fluorescence intensities, among which the fluorescence expression level of the 3DF‐LH group was the lowest. The results indicated that 3DF‐LH could significantly inhibit the expression of TGF‐β1, IL‐6, and p‐Smad2/3.

Smad2 and Smad3 serve as canonical downstream effectors of the TGF‐β1‐activated signaling cascade.^[^ ^40^ ^]^ Upon ligand‐induced phosphorylation, these Smad proteins heterodimerize with Smad4 to form transcriptional complexes that translocate to the nucleus, directly regulating fibrogenic gene expression through sequence‐specific DNA binding and chromatin remodeling.^[^ ^41^ ^]^ Notably, Smad7 functions as a potent negative regulator of this pathway by competitively inhibiting the formation of Smad2/3‐Smad4 heteromeric complexes, thereby blocking nuclear translocation and subsequent transcriptional activation of profibrotic targets.^[^ ^42^ ^]^ The phosphorylation levels of Smad2/3 directly correlate with fibrotic progression severity.^[^ ^43^ ^]^ Pathological collagen deposition‐ mediated predominantly by type I (COL1A1) and type III (COL3A1) collagen isoforms—constitutes a hallmark of extracellular matrix (ECM) remodeling during adhesion formation, where elevated collagen content directly correlates with adhesion severity.^[^ ^44^, ^45^ ^]^ To mechanistically validate the transcriptional regulation of fibrotic pathways, quantitative real‐time PCR (qRT‐PCR) was performed in accordance with to quantify mRNA transcript levels of Smad7, COL1A1, and COL3A1 across experimental groups (Figure 6c–e). Compared with the control, 3DF, and 3DF‐H groups, the expressions of COL1A1 and COL3A1 were significantly decreased in the Interceed and 3DF‐LH groups, with the most pronounced reduction observed in the 3DF‐LH group. Smad7 expression was significantly pronounced in the 3DF‐LH group compared to the control, 3DF, 3DF‐H, and Interceed groups. The results indicated that 3DF‐LH inhibited abdominal adhesions by regulating the phosphorylation of the TGF‐β1/Smad signaling pathway and reducing the formation of collagen.

## 3 Conclusion

In this work, inspired by the framework‐reinforced structure and construction process of concrete, we developed a 3D‐printed PCL framework/PSBMA hydrogel composite patch with proper mechanical strength and lubricative performance, which effectively addresses the fragile problem of PSBMA hydrogel in the application of postoperative adhesion prevention. Upon incorporation of the 3D‐printed framework, the elongation at break of 3DF‐LH increased from 27.34 ± 6.31% to 205.14 ± 3.23%, and its tensile strength improved from 0.07 ± 0.01 to 4.43 ± 0.14 MPa. These enhanced mechanical properties enable the zwitterionic hydrogel to be securely sutured during surgical procedures, preventing rupture due to mechanical weakness and providing reliable barrier functionality. Furthermore, 3DF‐LH outperforms the commercial Interceed in attenuating the inflammatory microenvironment through potent inhibition of the TGF‐β/Smad signaling pathway, thereby more effectively suppressing adhesion formation. Notably, 3DF‐LH exhibits excellent biocompatibility. Our findings propose an effective, concrete‐inspired strategy for reducing postoperative adhesions, highlighting its significant potential for clinical translation.

## 4 Experimental Section

### Materials

PCL and allyl methacrylate were obtained from Rhawn Chemical Technology Co., Ltd. (Shanghai, China). Photoinitiator I2959, gelatin, GelMA, and SBMA were sourced from Yuanye Bio‐Technology Co., Ltd. (Shanghai, China). Reagents used in the cell culture experiments and antibodies were purchased from Service (Wuhan, China).

### Cells

The mouse embryonic fibroblast cell line (NIH/3T3) was purchased from Wuhan Service Biotechnology Co., Ltd.

### Animals

Male SD rats (220 ± 20 g) were obtained from the Shandong Xinbainuo Biotechnology Co., Ltd. All animal procedures were conducted at the Experimental Animal Center of the Affiliated Hospital of Hebei University under protocols approved by the Institutional Animal Care and Use Committee (20 240 302).

### Fabrication of 3D‐Printed Frameworks

PCL frameworks were fabricated using fused deposition modeling (FDM) technology (SunP BioMaker 2, SunPbiotech, China). PCL granules (Mw = 80 kDa) were loaded into a preheated extrusion barrel at 80 °C under a controlled pressure range of 600–700 kPa. Printing parameters included a deposition speed of 5 mm s^−1^, an extrusion rate of 0.72 mm s^−1^, and a fiber diameter of 0.6 mm. The frameworks were designed with alternating layer orientations (0°/90°, 0°/45°/90°, and 0°/30°/60°), a strand spacing of 1 mm, and a layer thickness of 0.2 mm.

### Construction of Composite Frameworks

GelMA (5–15% w/v) and SBMA (1–3% w/v) solutions were blended, containing 0.5% (w/v) I2959 (relative to the total solution). The precursor solution was infiltrated into the porous architecture of 3D‐printed PCL frameworks. Photocrosslinking was achieved via 405 nm blue light irradiation (10 mW cm^−^^2^, 5 min), yielding a lubricious hydrogel‐integrated composite framework. Final constructs were sterilized by UV exposure.

### Material Characterization

Framework surface topography was examined using field‐emission scanning electron microscopy (FE‐SEM, Quanta200, FEI). Samples were sputter‐coated with a 10 nm platinum layer (EM ACE600, Leica) prior to imaging.

### Chemical Characterization

Fourier‐transform infrared spectroscopy (FTIR, Nicolet iS50, Thermo Fisher) was performed in transmission mode (400–4000, 4 cm^−1^ resolution, 32 scans) using KBr pellets (sample/KBr ratio 1:150 w/w).

### Tensile Tests

3DF with different stent architectures were prepared into dumbbell specimens (10 mm in length, 10 mm in width, and 1 mm in thickness) for tensile tests (n = 3). Tensile property measurements of hydrogels were performed using a universal mechanical testing machine (WD‐7A, Guangzhou Experimental Instrument Factory, China) with a 100 N load cell and an extension speed of 10 mm min^−1^.

### Compressive Tests

Compressive tests were performed on a universal mechanical testing machine (WD‐7A, Guangzhou Experimental Instrument Factory, China) at a compression speed of 5 mm min^−1^. The tested 3DF was cylindrical of 5 mm in height and 10 mm in diameter. Compressive strength of the 3DF was determined by measuring the ratio of the maximum force required to crush the hydrogel to the contact area.

### Evaluation of Swelling Behavior

To evaluate the swelling behavior, 3DF‐H and 3DF‐LH samples with 10 mm in diameter and 3 mm in height were immersed in 5 mL of phosphate‐buffered saline solution (PBS; pH = 7.4) at 37 °C (n = 3). At each predetermined time point, the immersed samples were taken out and weighed. The swelling ratio was calculated by the following equation: Swelling ratio (%) = (w_t_ – w_0_)/ w_0_ × 100%. Where w_0_ is the weight of the original dry hydrogels, and w_t_ is the weight of the swollen hydrogels at different time points.

### Water Vapor Transmission Test

The moisture permeability of 3DF‐LH and Tegaderm was determined using the ASTM E96 standard (n = 3). The samples were cut into 74 mm diameter circles and placed in a cylindrical moisture‐permeable cup containing 10 mL of DI water. These parts were sealed and tested at 37 °C and 90% relative humidity. Every hour, the cup was weighed and recorded until the readings were steady. The experiment was repeated three times, and the average result was obtained. Water Vapor Transmission Rate (WVTR) was calculated as: WVTR = (24 × Δm) / (A × t). Δm = mass change over stable interval (g), A = specimen area (π × r^2^ = 0.0043 m^2^), t = time interval between measurements (h), values are expressed in g·m^−2^·h^−1^.

### Measurement of Oxygen Permeability Coefficient (OPC)

OPC of 3DF‐LH and Tegaderm was determined according to ASTM D3985 using a coulometric oxygen permeation analyzer (Createch 201T Oxygen Permeameter). After being fully hydrated in PBS solution (pH 7.2), the materials were measured to get their center thickness (n = 3). Then, these samples were put on the electrode successively, and polarography was used to measure the electric current values. The temperature should be controlled at 35 ± 5 °C. The air humidity should be controlled at ≈95%. Finally, we calculated the slope of the center thickness–electric current value curve to get the OPC value. The measured values of oxygen permeability were expressed in terms of the barrier.

### Degradation Profiling

The materials were thoroughly dried in a vacuum oven before being cut into 1 × 1 cm squares and weighed (designated as W_i_). For 14 days, the weighed samples were immersed in 20 ml of PBS containing 10 mg of collagenase II at 37 °C (n = 3). The samples were withdrawn from the PBS solution at each time point, washed, and weighed (W_d_) after vacuum drying. The weight losses were computed using the equation below: Degradation rate = (W_i_‐ W_d_)/ W_i_ × 100%

### Antibacterial Activity Tests

Antibacterial activity of 3DF‐LH toward *S. aureus* and *E. coli* was evaluated using colony‐forming units (CFU). Specifically, 3DF, 3DF‐H, 3DF‐LH (size: 10 mm ×10 mm× 1 mm) and PBS were co‐cultured with bacteria at 37 °C, respectively. Then, 100 µL of the bacterial solution extracted from the surface of each material (or from the PBS control) was uniformly spread on agar plates, which were cultured in the incubator for 14 h at 37 °C. Bacterial colony forming units were photographed to evaluate the inhibitory effect.

### Live/dead Staining

Antibacterial efficacy was further assessed using SYTO‐9 (green‐fluorescent) and propidium iodide (PI; red‐fluorescent) live/dead staining. Bacteria were incubated for 6 h with Blank (PBS‐treated), 3DF, 3DF‐H, and 3DF‐LH, respectively. The stained bacteria were then imaged using fluorescence microscopy (n = 3).

### Bacterial Morphological Characterization

Bacterial morphology was examined using scanning electron microscopy (SEM; Quanta 200, FEI). Briefly, bacteria were incubated for 6 h with PBS (Blank), 3DF, 3DF‐H, and 3DF‐LH. Samples were then fixed in 2.5% glutaraldehyde for 2 h, dehydrated through a graded ethanol series, and critical‐point dried prior to SEM observation (n = 3).

### In Vivo Hemostasis Ability on Rat Livers

All animal procedures were performed under general anesthesia (n = 3), which was maintained using propofol (0.2 mg kg^−1^ min^−1^). The liver was exposed, and a pre‐weighed filter paper was placed beneath the liver. A liver incision (length: 1.5 cm, depth: 0.3 cm) was made. 3DF, 3DF‐H, 3DF‐LH were applied to the bleeding site. The blood loss was recorded until hemostasis was achieved.

### Blood Clotting Index

The BCI evaluated the potential to stimulate in vitro blood coagulation. CaCl_2_ (1 mL, 0.1 m) was added to heparinized rat blood (9 mL) to activate the blood. 50 µL activated blood was put onto the hydrogels, and 5 mL of deionized water was used to dissolve the uncoagulated blood. UV–vis spectroscopy was used to measure OD at 540 nm. BCI (%) = OD _sample_/ OD _reference_ × 100%. The reference was 50 µL of blood in 5 mL of deionized water. Each sample was repeated three times.

### Cytocompatibility

Cytotoxicity was evaluated using both direct contact and leaching assays (n = 3). In the direct contact assay, designed to assess the effect of GelMA concentration on cell viability, materials with varying GelMA concentrations were prepared. NIH/3T3 cells (1 × 10^5^ cells mL^−1^) were directly seeded onto the material surfaces in 24‐well plates (100 µL per well) and incubated at 37 °C with 5% CO_2_ for 1, 3, or 7 days. For the indirect contact (leaching) assay, assessing 3DF‐LH cytotoxicity, materials were first incubated in serum‐containing medium (0.1 g mL^−1^) at 37 °C for 24 h to prepare extracts. Separately, NIH/3T3 cells (1 × 10⁵ cells mL ^−1^) were seeded into empty wells of 24‐well plates (100 µL per well), cultured for 24 h to allow attachment, and then the medium was replaced with 100 µL of material extract (experimental groups) or 100 µL of fresh DMEM (control group), followed by incubation for 1, 3, or 7 days at 37 °C with 5% CO_2_. For both methods, at each time point (1, 3, 7 days), cell viability was assessed: (1) using LIVE/DEAD staining reagents applied for 10 min followed by fluorescence imaging; and (2) via CCK‐8 assay, where the medium was replaced and cells incubated for a further 24 h before adding fresh DMEM containing 10 µL CCK‐8 solution (1 mg mL^−1^), incubating for 2 h, and measuring absorbance at 450 nm. Cell viability was calculated using the following formula: Cell viability (%) = [(OD _sample_ – OD _blank_) / (OD _control_ – OD _blank_)] × 100%.

### Hemocompatibility

The hemolytic activity of the 3DF, 3DF‐H, and 3DF‐LH was determined using a hemolysis assay. RBCs were isolated from heparinized horse whole blood samples by centrifugation and washed three times with PBS. The RBCs were diluted to a final concentration of 5% (v/v) in PBS. Materials (0.2 g) were incubated with 2 mL of RBC suspension at 37 °C for 1 h. After incubation, the samples were centrifuged at 3000 rpm for 10 min, and the OD of the supernatant was measured at 540 nm (n = 3). The hemolysis ratio was then calculated as follows: Hemolysis ratio (%) = [(OD _sample_ – OD _0.9%NaCl_) / (OD _TritonX‐100_ – OD _0.9%NaCl_)] × 100%.

### Anti‐Cell Adhesion Evaluation

The cell adhesion performance of 3DF‐LH was evaluated via direct contact assay using NIH/3T3 fibroblasts (1 × 10^5^ cells well^−1^). Prior to cell seeding, 24‐well plates were coated with Matrigel (Corning, Cat# 354 230) by diluting thawed matrix 1:20 in serum‐free DMEM (final concentration: 500 µg mL^−1^), adding 500 µL well^−1^, and polymerizing at 37 °C for 1 h. Unbound matrix was aspirated, followed by two PBS washes. Cells were then seeded onto 3DF, 3DF‐H, and 3DF‐LH substrates and incubated at 37 °C/5% CO_2_ for 1, 3, and 7 days. At each timepoint, non‐adherent cells were removed through gentle aspiration and three sequential PBS rinses (pre‐warmed, dispensed along well walls, 10‐s incubation, diagonal aspiration). Adherent cells were fixed with 4% paraformaldehyde, permeabilized with 0.1% Triton X‐100, and stained with Alexa Fluor 488‐phalloidin and DAPI for cytoskeletal and nuclear visualization. Fluorescence images were acquired using an inverted microscope (Nikon Eclipse Ti2), with cell adhesion area quantified from five random fields/well via ImageJ v1.53 (FIJI) under blinded analysis. Biological replicates comprised three independent experiments with triplicate wells per group (n = 9).

### Anti‐Protein Adsorption Assay

Protein adsorption was measured via bicinchoninic acid (BCA) assay (n = 3). Hydrogel discs (Φ6 mm) were incubated with bovine serum albumin (1 mg mL^−1^ PBS), followed by elution with 1% sodium dodecyl sulfate (SDS). Absorbance at 562 nm was correlated with standard curves to determine adsorbed protein mass (µg cm^−2^).

### Biomechanical Simulation Testing

A porcine skin model (3 × 8 cm^2^) was sutured with hydrogel samples (4‐0 Prolene) and subjected to cyclic tensile loading (15% strain, 0.5 Hz) using surgical instruments. Structural integrity was monitored throughout 10 loading cycles (n = 3).

### In Vivo Anti‐Adhesion Evaluation

A standardized cecal‐abdominal wall abrasion model was established in male Sprague‐Dawley rats (n = 3). Surgical procedures involved preoperative fasting for 24 h, followed by anesthesia induction and maintenance via isoflurane inhalation. After abdominal hair removal and aseptic preparation, a midline laparotomy was performed using sterile surgical scissors. The cecal serosa was mechanically abraded with sterile gauze until punctate hemorrhage appeared, while a 1 × 1 cm abrasion was created on the adjacent abdominal wall (Figure S7, Supporting Information). The injured cecum was exposed to the abdominal wall injury site and secured with 4‐0 non‐absorbable polypropylene sutures to ensure adhesion formation. Animals were randomly divided into five groups: Control, 3DF, 3DF‐H, Interceed, and 3DF‐LH. Implants were secured at the four corners and midpoint of the scaffold using 6‐0 non‐absorbable polypropylene sutures over the defect site. Following cecal repositioning, the abdominal wall and skin were closed in layers with 4‐0 chromic gut and nylon sutures, respectively. Postoperative analgesia was administered via subcutaneous buprenorphine (1 mg kg^−1^), and animals were monitored daily for signs of infection or dehiscence until scheduled euthanasia timepoints.

### Outcome Measures

Adhesion severity was graded in accordance with established criteria in the literature at postoperative days 7 and 14 (Table S2, Supporting Information). Adhesion tissues were harvested from euthanized rats at postoperative days 7 and 14, sectioned for histological processing, and subjected to hematoxylin and eosin (H&E) staining alongside Masson's trichrome staining to evaluate connective tissue hyperplasia and collagen deposition patterns. Molecular mechanisms were investigated via immunofluorescence (TGF‐β1, IL‐6 and P‐Smad2/3), and qRT‐PCR (COL1A1, COL3A1, Smad7).

### In Vivo Biosafety Evaluation

At 14 days post‐intraperitoneal implantation, rats were euthanized, and abdominal surgical suture site tissues, along with major organs (heart, liver, spleen, lungs, and kidneys), were harvested. All specimens were fixed in 10% neutral buffered formalin for 48 h, embedded in paraffin, sectioned at 5‐µm thickness, and subjected to H&E staining for histopathological biocompatibility assessment. Concurrently, hematological and biochemical analyses were performed on blood samples collected at the same time point (14 days post‐implantation). The following parameters were analyzed: aspartate aminotransferase (AST), alanine aminotransferase (ALT), total protein (TP), albumin (ALB), blood urea nitrogen (BUN), creatinine (Crea), red blood cell (RBC) count, white blood cell (WBC) count, and platelet (PLT) count.

### Statistical Analysis

Data were presented as mean ± standard deviations (SDs). Student's t‐test or one‐way ANOVA was used to assess the difference between two groups or multiple groups using GraphPad Prism software, respectively. The levels of significance were labeled with ^*^ *p* < 0.05, ^**^ *p* < 0.01, ^***^ *p* < 0.001, and ^****^ *p* < 0.0001.

## Conflict of Interest

The authors declare no conflict of interest.

## Supporting information



Supporting Information

## Data Availability

The data that support the findings of this study are available from the corresponding author upon reasonable request.
