# Supplementary material for: Concrete Structure Inspired 3D‐Printed Framework Mechanically Reinforced Zwitterionic Hydrogel for Efficient Postoperative Abdominal Adhesion Prevention
Source: Adv Sci (Weinh). 2025 Sep 16;12(45):e08954. doi: 10.1002/advs.202508954 (PMC12677669; doi:10.1002/advs.202508954)
Supplement: Supplementary file 1 — Supporting Information [file ADVS-12-e08954-s001.docx]

**Supporting Information**

Concrete Structure Inspired *3D Printed* Framework Mechanically Reinforced Zwitterionic Hydrogel for Efficient Postoperative Abnormal Adhesion Prevention

Jianguo Song, ^a, b 1^ Xin Cao, ^a, b 1^ Ruirui Zhang, ^a, b^, Shujie Cheng, ^b, *^ Yi Wang ^a, b, *^

^a^ College of Mechanical & Energy Engineering, Beijing University of Technology, Beijing 100124, China.

^b^ Basic Research Key Laboratory of General Surgery for Digital Medicine, Affiliated Hospital of Hebei University, Baoding 071000, China.

^1^ Co-first authors.

^*^ Corresponding authors.

E-mail addresses:

wangyi2022@bjut.edu.cn (Prof. Y. Wang),

chengshujiehbu@163.com (Prof. S.J. Cheng)

**Table S1.** Blood biochemistry and routine hematological indicators on day14

|  | **Group** | | | | **Reference Range** |
| --- | --- | --- | --- | --- | --- |
|  | Control | 3DF | 3DF-H | 3DF-LH |  |
| AST (U/L) | 108 ± 6 | 94± 9 | 140 ± 12 | 121 ± 21 | 84 – 152 |
| ALT (U/L) | 29 ± 2 | 33 ± 3 | 35 ± 3 | 39 ± 3 | 25– 44 |
| TP (g/L) | 55 ± 2 | 61 ± 3 | 60 ± 2 | 64 ± 3 | 53 – 70 |
| ALB (g/L) | 28 ± 1 | 33± 1 | 30 ± 2 | 32 ± 2 | 24 – 36 |
| A/G | 1.1 ± 0.1 | 1.1 ± 0.1 | 1.0 ± 0.1 | 1.0 ± 0.1 | 0.6 – 1.2 |
| BUN (mmol/L) | 5± 2 | 8 ± 2 | 7 ± 1 | 9 ± 2 | 3–11 |
| Crea (umol/L) | 18 ± 2 | 33 ±3 | 35 ± 2 | 39± 5 | 16 – 44 |
| RBC (*10^12^ /L) | 6 ± 1 | 5 ± 1 | 8 ± 1 | 6 ± 1 | 4 – 10 |
| WBC (*10^9^ /L) | 8 ± 3 | 7 ± 1 | 8 ± 1 | 6 ± 2 | 4 – 12 |
| PLT (*10^9^ /L) | 1036 ± 124 | 1035 ± 88 | 1191 ± 21 | 1129 ± 105 | 737 –1342 |

AST: Aspartate aminotransferase, ALT: Alanine aminotransferase, TP: Total protein, ALB: Albumin, A/B: Albumin globulin ratio, BUN: Blood urea nitrogen, Crea: Creatinine, RBC: Red blood cells, WBC: White blood cells, PLT: Platelets

**Table S2.** Site-Specific classification of adhesions

| Adhesion characteristics | Score |
| --- | --- |
|  |  |
| Extent of site involvement |  |
| None | 0 |
| <25% | 1 |
| <50% | 2 |
| <75% | 3 |
| <100% | 4 |
|  |  |
| Type |  |
| None | 0 |
| Filmy, transparent, avascular | 1 |
| Opaque, translucent, avascular | 2 |
| Opaque, capillaries present | 3 |
| Opaque, larger vessels present | 4 |
|  |  |
| Tenacity |  |
| None | 0 |
| Adhesion falls apart | 1 |
| Adhesion lysed with traction | 2 |
| Adhesion requiring sharp dissection | 3 |
| Possible total | 11 |


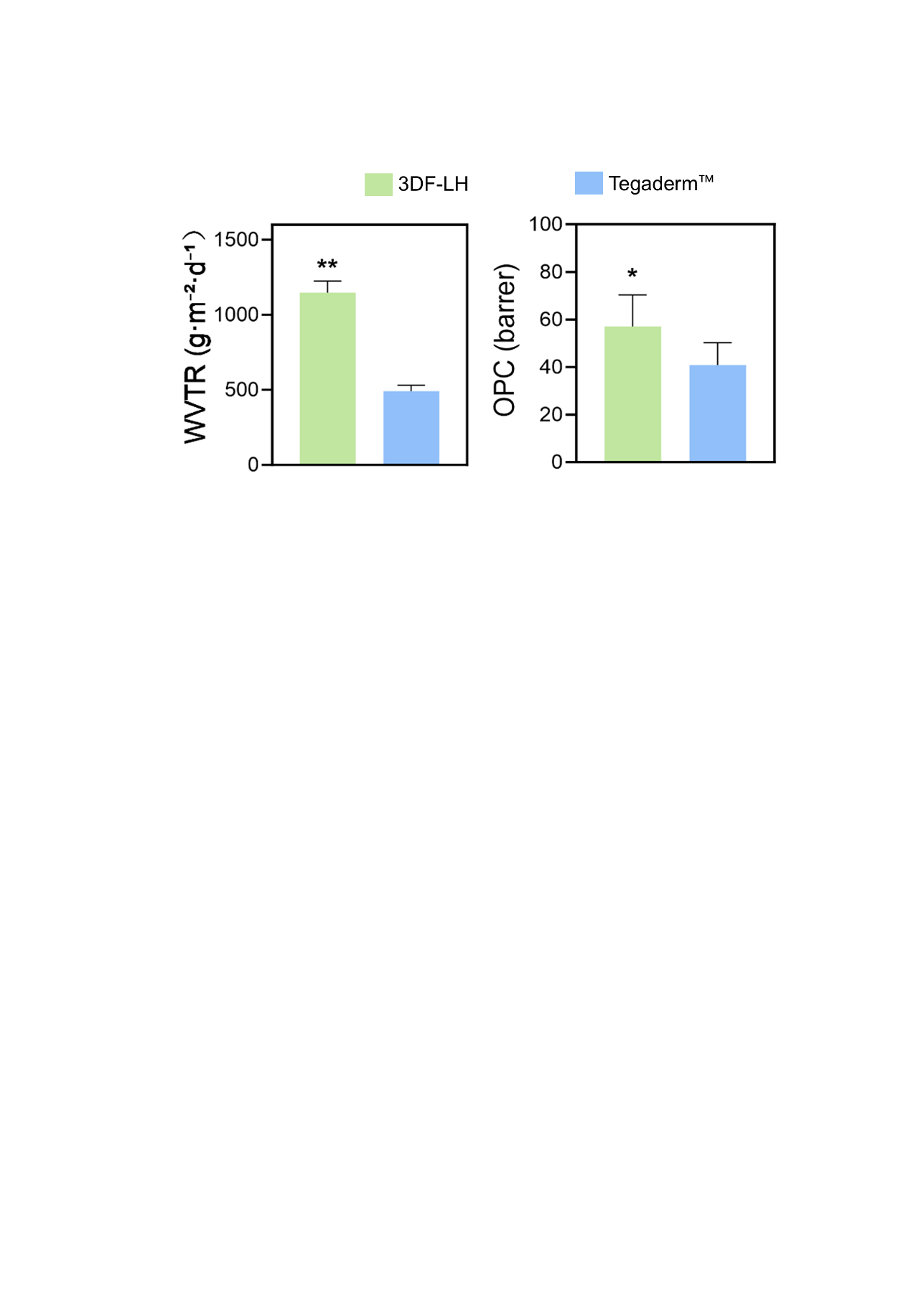


**Figure S1.** WVTR and OPC of different samples. 1 barrer equals 7.5 × 10^-18^m^3^·m^-1^·s^-1^·Pa^-1^. Data are presented as mean ± SDs, *p < 0.05, **p < 0.01, compared to the Tegaderm^TM^ group.


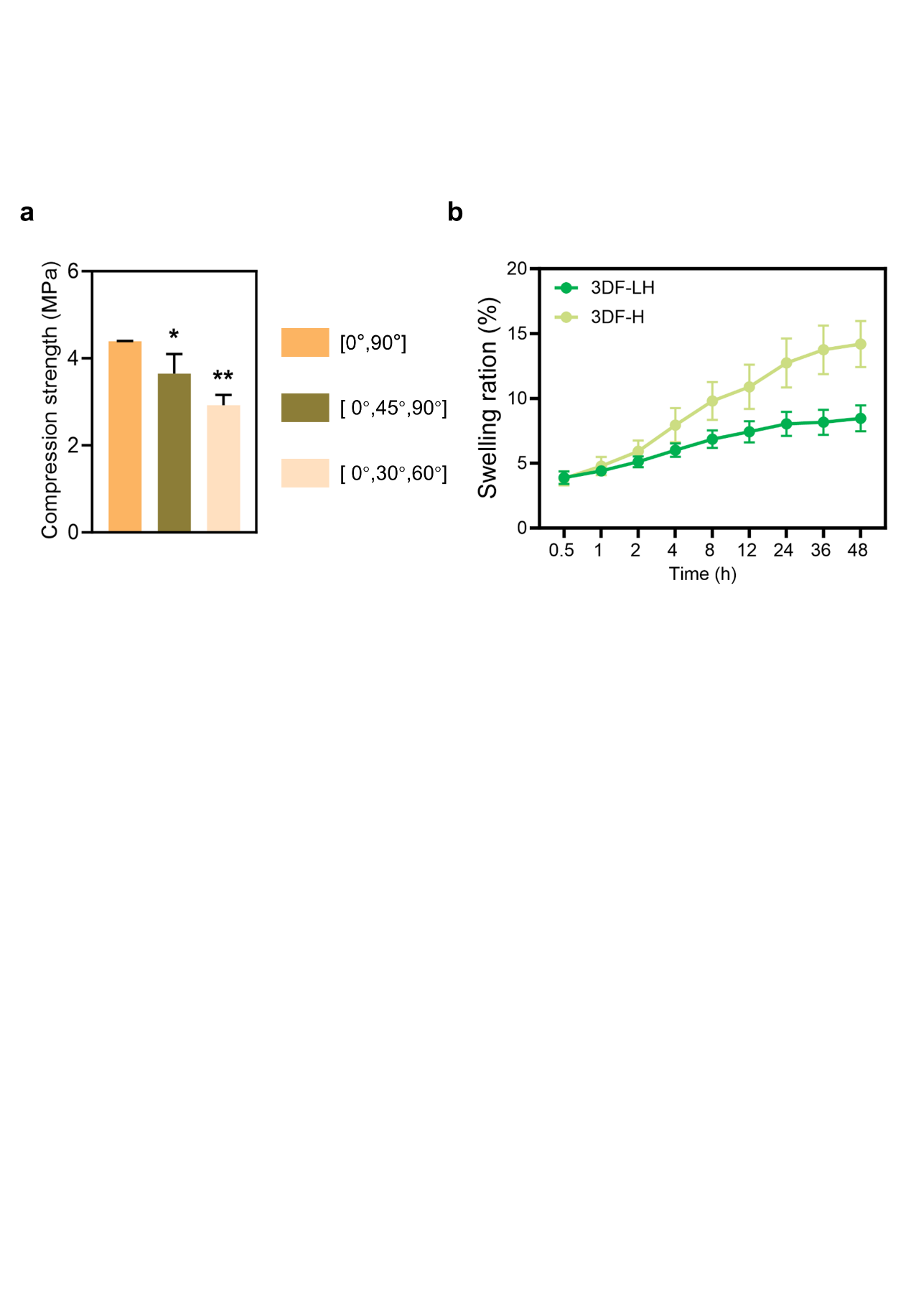


**Figure S2.** (a) Compressive strength of diverse stent architectures. (b) Swelling kinetics curves of hydrogels in PBS (pH=7.4) at 37 °C. Data are presented as mean ± SDs, *p < 0.05, **p<0.01, compared to the 0°-90° group.


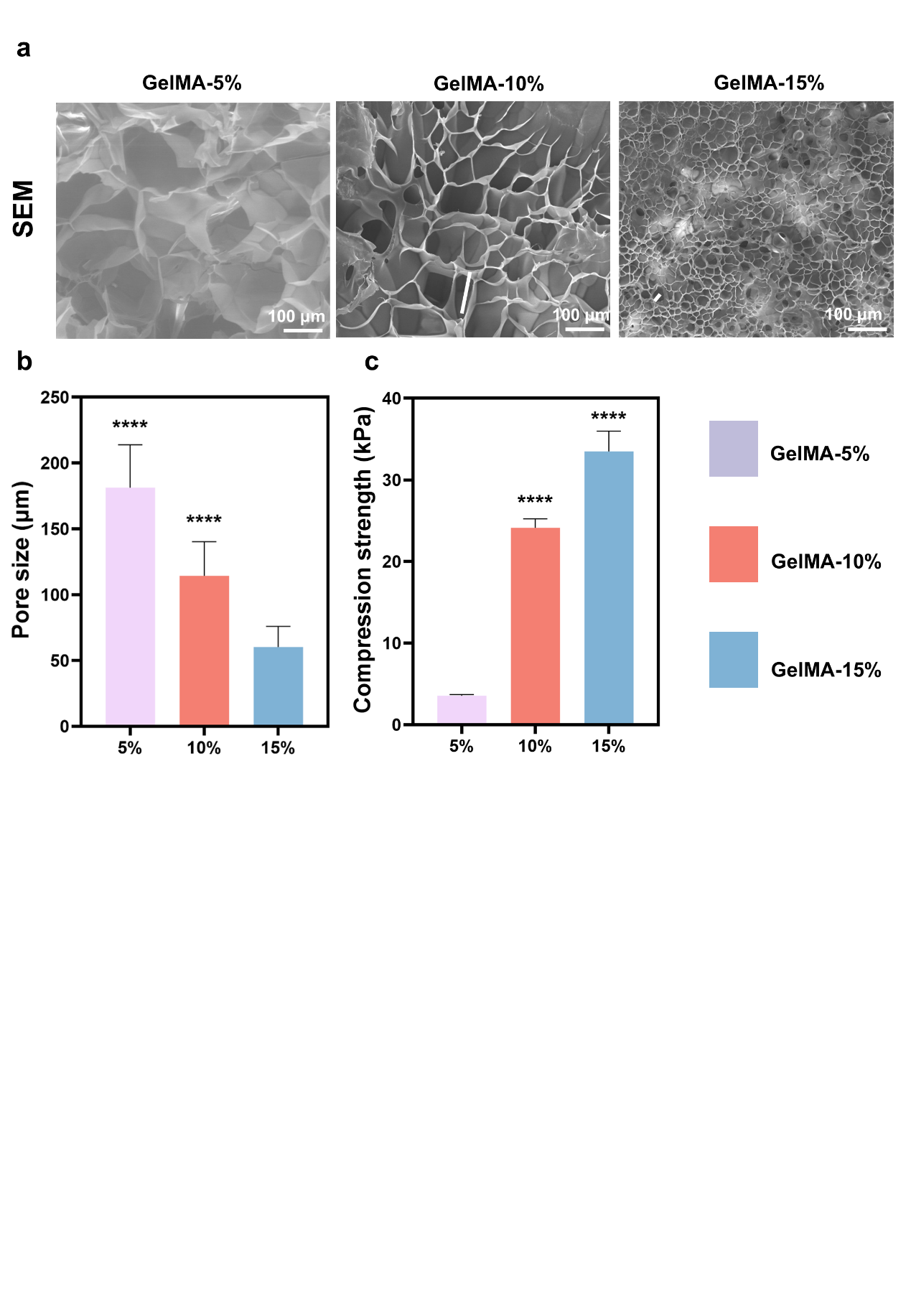


**Figure S3.** (a) SEM images, (b) pore size distributions, and (c) compressive strength of GelMA hydrogels at varied concentrations. Data are presented as mean ± SDs, ***p < 0.001, ****p<0.0001, compared to the 15% GelMA hydrogel group.

**
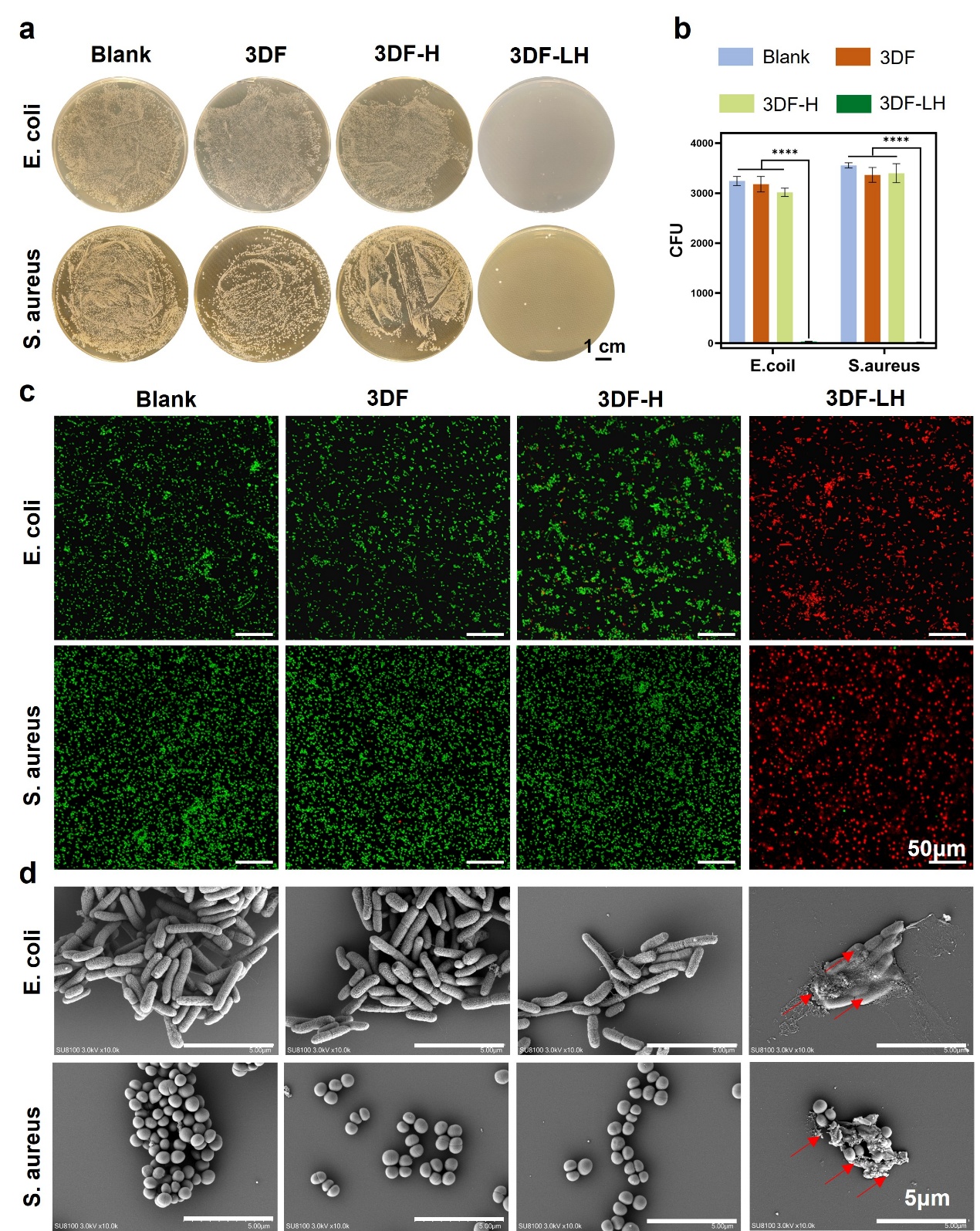
**

**Figure S4**. (a) Bacteria colonies images of *E. coli* and *S. aureus* on Blank, 3DF, 3DF-H, and 3DF-LH. (b) Bacterial survival ratio of *E. coli* and *S. aureus* on Blank, 3DF, 3DF-H, and 3DF-LH. (c) Live/dead staining images of *E. coli* and *S. aureus* cultured with PBS, 3DF, 3DF-H, and 3DF-LH. (d) SEM images of *E. coli* and *S. aureus* cultured with PBS, 3DF, 3DF-H, and 3DF-LH. Cell membrane lysis indicated by red arrows. Data are presented as mean ± SDs, ****p<0.0001, compared to 3DF-LH group.

**
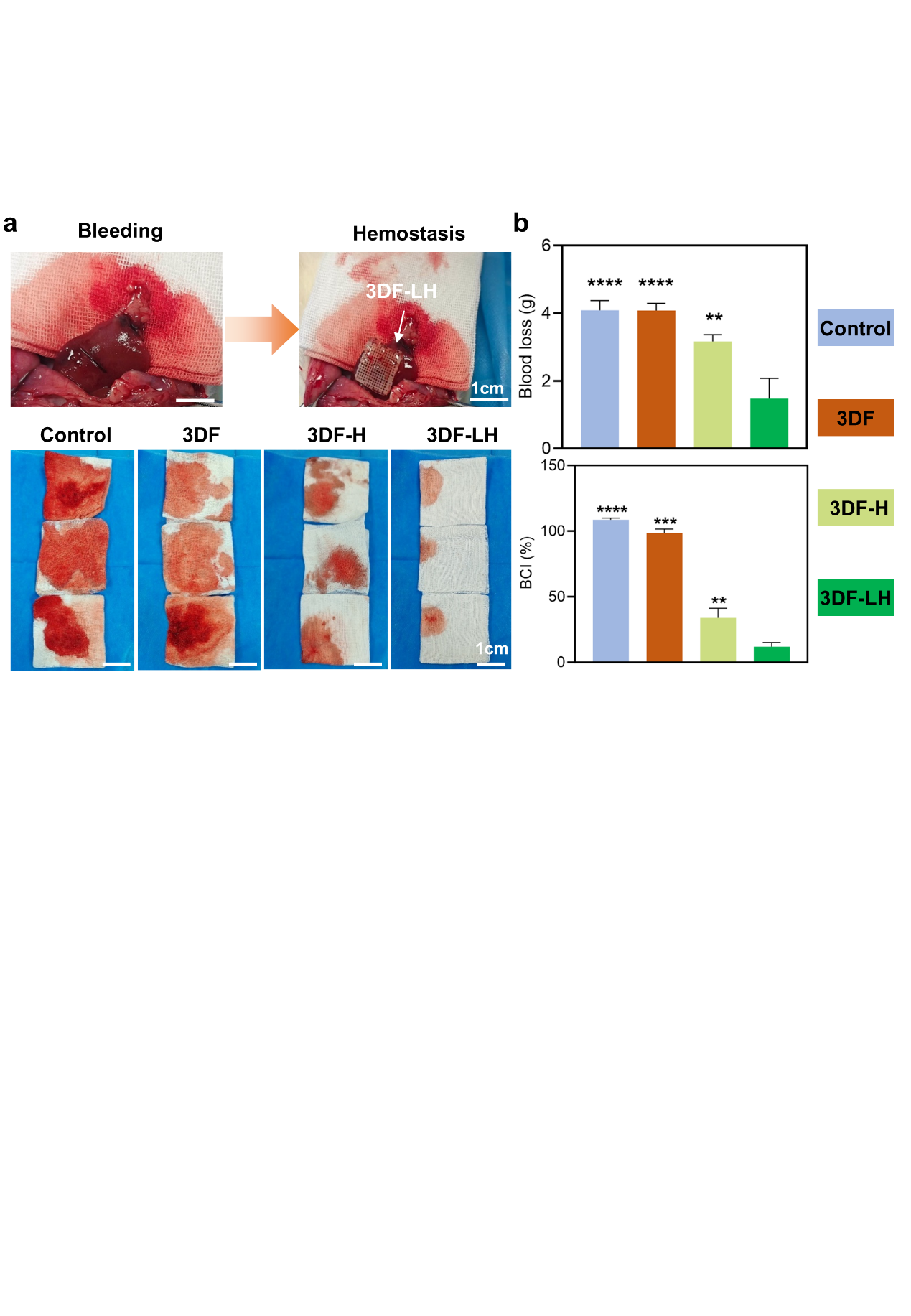
**

**Figure S5.** (a) Established SD rat liver injury model, and the dynamic hemostatic ability using either 3DF, 3DF-H and 3DF-LH. (b) Quantitative analysis of blood loss and blood clotting index (BCI) of control, 3DF, 3DF-H and 3DF-LH groups. Data are presented as mean ± SDs, **p < 0.01, ***p<0.001, ****p<0.0001, compared to the 3DF-LH group.


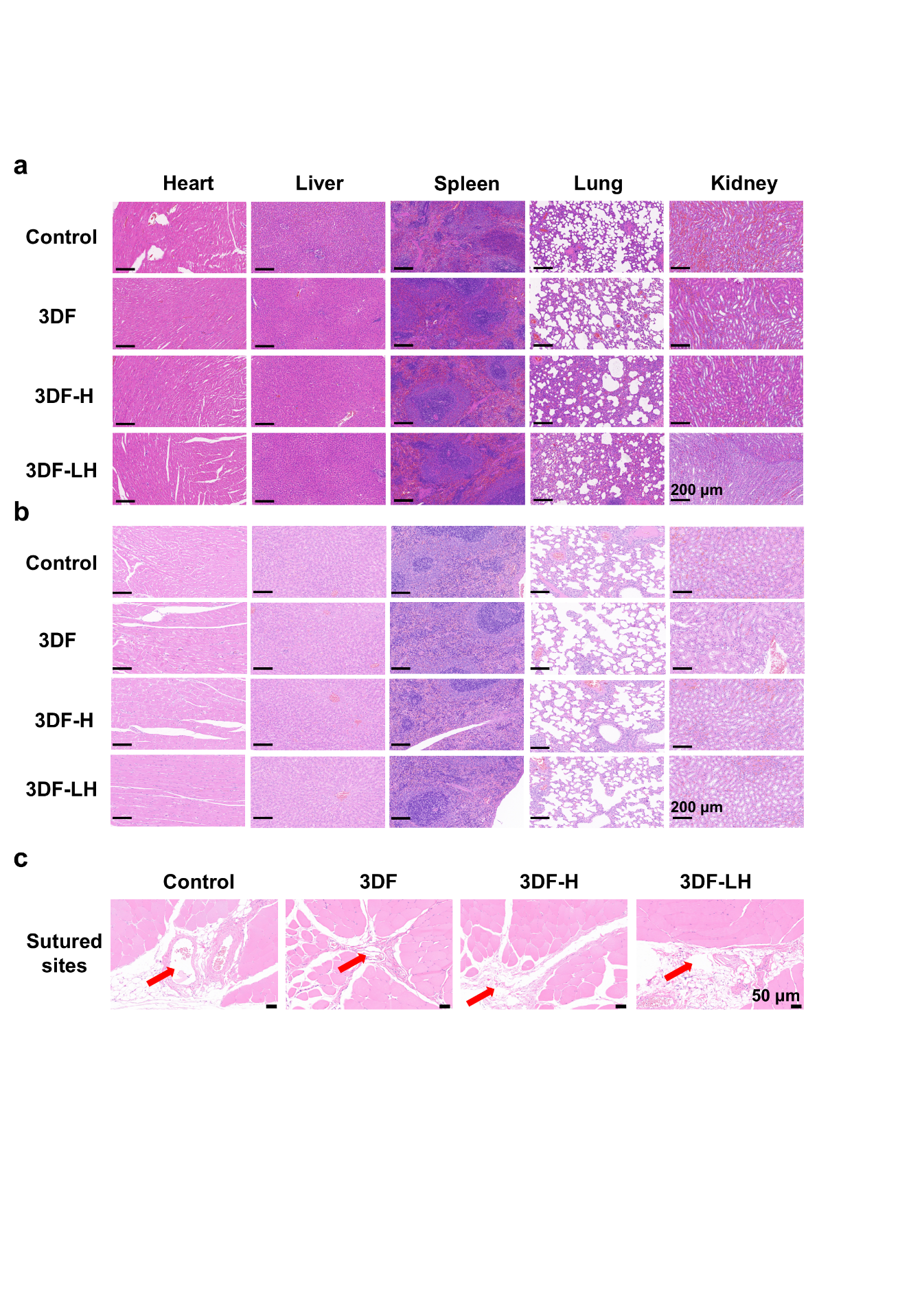


**Figure S6.** (a) H&E staining images of major organs harvested at day 14 post-subcutaneous implantation. (b) H&E staining images of major organs harvested at day 14 post-intraperitoneal implantation. (c) H&E staining images of the sutured sites at day 14 post-surgery.


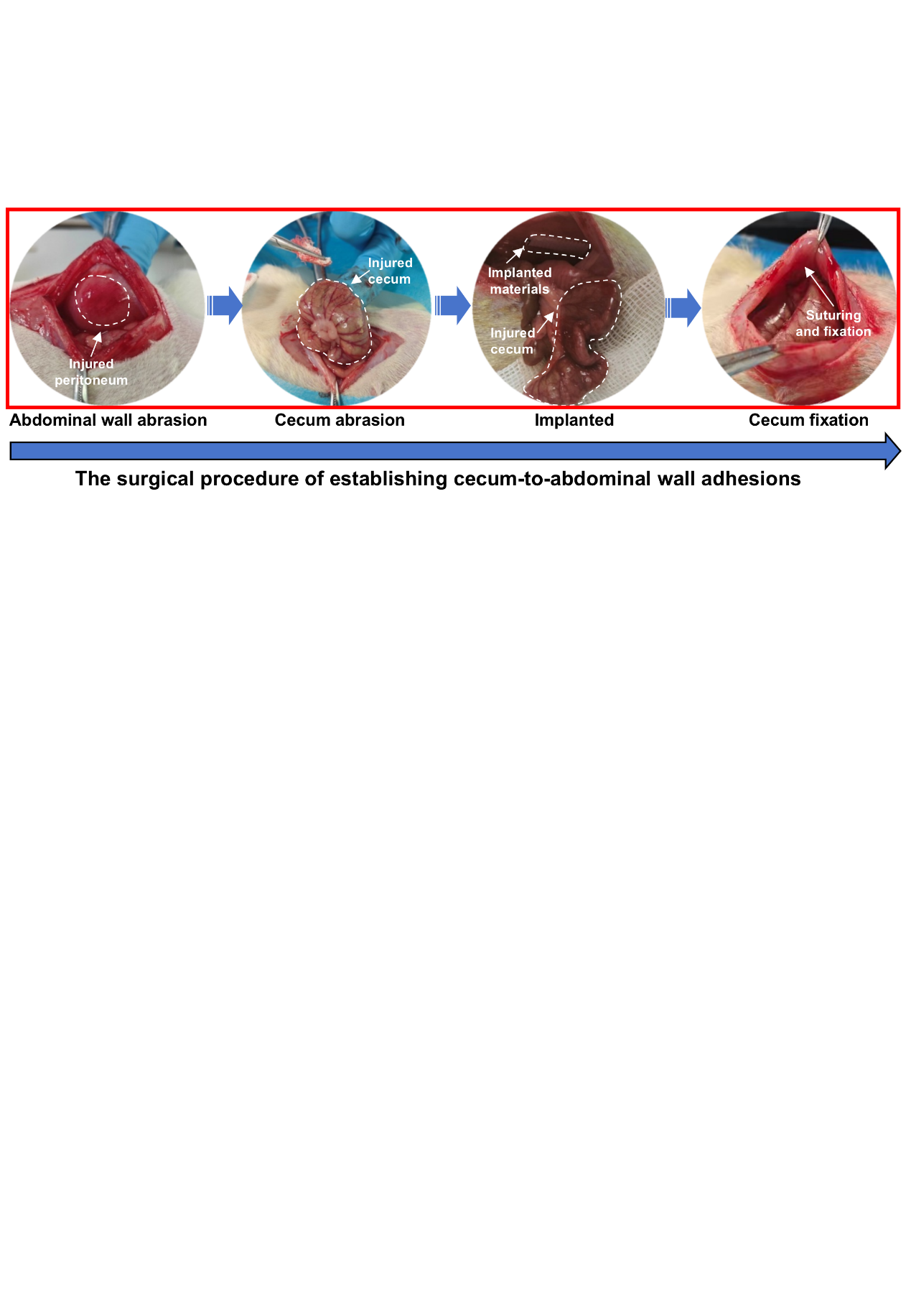


**Figure S7.** Surgical induction of cecal-abdominal wall adhesions in rat model.
